# Compatibility of a novel temperature‐controlled, irrigated radiofrequency catheter with ultra‐high‐density mapping

**DOI:** 10.1002/joa3.12896

**Published:** 2023-07-08

**Authors:** Daniel Rodriguez Muñoz, Álvaro Marco del Castillo, Javier Ramos Jimenez, Luis Borrego Bernabe, Alba Madrid Montoya, Adrián Lorenzo Balboa, Fernando Arribas Ynsaurriaga, Rafael Salguero‐Bodes

**Affiliations:** ^1^ Cardiology Department University Hospital 12 de Octubre and Instituto de Investigación Sanitaria Hospital 12 de Octubre (imas12) Madrid Spain; ^2^ Rhythmia HDx, Boston Scientific Marlborough Massachusetts USA; ^3^ Medtronic, Inc. Minneapolis Minnesota USA; ^4^ Centro de Investigación Biomédica en Red de enfermedades CardioVasculares (CIBERCV), Instituto de Salud Carlos III Madrid Spain; ^5^ Department of Medicine Universidad Complutense Medical School Madrid Spain

**Keywords:** cardiac mapping, DiamondTemp, Rhythmia, temperature‐controlled ablation, ultra‐high‐density

## Abstract

**Background:**

Compatibility of DiamondTemp (DT) radiofrequency (RF) catheter with the Rhythmia mapping system has not been manufacturer‐reported nor its tracking accuracy reported.

**Methods:**

Consecutive patients undergoing macroreentrant atrial tachycardia ablation guided by Rhythmia and ablated using DT were prospectively enrolled. Following catheter configuration, ablation lines were performed and remapped to measure the RF tag to effective‐ablation‐line‐center (RFT‐ALC) distance.

**Results:**

Among 20 consecutive patients (54 maps), 40 ablation lines were evaluated. Overall, the RFT‐ALC distance was 3.88 ± 2.95 mm, and the operator assessment of accuracy was high. No complications occurred.

**Conclusion:**

The use of DT catheter guided by the Rhythmia mapping system is feasible and accurate.

## Graphical abstract

Compatibility of DiamondTemp (DT) radiofrequency (RF) catheter with the Rhythmia mapping system has not been reported. Consecutive patients undergoing macroreentrant atrial tachycardia using DT and Rhythmia were included, proving this combination to be feasible and showing good accuracy results.

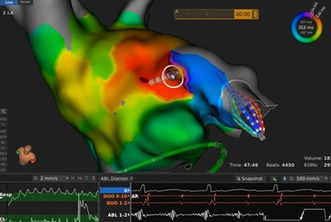

## 1 INTRODUCTION

The DiamondTemp (DT) ablation catheter (Medtronic, Inc.) uses industrial diamond‐mediated rapid thermal diffusion and six external, tissue‐faced insulated thermocouples on the ablation tip to measure tissue surface temperature during radiofrequency (RF) ablation.^1^, ^2^ Reported data on the use of this catheter are still scarce, and electroanatomic mapping (EAM) compatibility has been limited to Ensite Precision (Abbott Medical, Inc.).^2^, ^3^ In parallel, the difference in the accuracy of catheter display between catheters with or without magnetic sensors is unknown.

The aim of this study was to explore the safety and accuracy of this catheter when used under the guidance of the Rhythmia HDX mapping system (Boston Scientific) in patients undergoing macroreentrant atrial tachycardia (MRAT) ablation. We considered this compatibility relevant given the advantages of ultra‐high‐density mapping with the Rhythmia mapping system, and potential benefits of the accurate tip–tissue interface temperature control of the DT, especially considering recent data showing a higher incidence of cerebral lesions with other catheters when used in high‐power short‐duration modes.^2^, ^3^, ^4^, ^5^, ^6^

We hypothesized that following adequate configuration, the catheter would be accurately displayed on the mapping system, enabling precise lesion placement with no workflow impairment.

## 2 METHODS

### 2.1 Study design

Consecutive patients undergoing MRAT were prospectively enrolled between June 2020 and January 2021. Electrophysiological studies were performed under general anesthesia. Ultra‐high‐density maps were created using a 64‐pole basket mapping catheter (IntellaMap Orion; Boston Scientific).

### 2.2 Mapping system configuration to enable catheter visualization

To enable DT catheter identification, a tripolar catheter of 7.5 Fr thickness was configured in the Rhythmia HDx mapping system. The composite RF electrode, which is split for higher electrogram resolution in a distal 0.6 mm tip and a proximal 3 mm ring separated by a 0.5 mm distal diamond, was defined as a single electrode. Sequentially, the third and fourth rings in the catheter were assigned to the middle and proximal electrodes in the EAM system. This adjustment was adopted since the attempts to reproduce the split‐tip configuration yielded errors in impedance measurement and catheter visualization. Interelectrode spacing was defined as 3.5–2.

**FIGURE 1:**
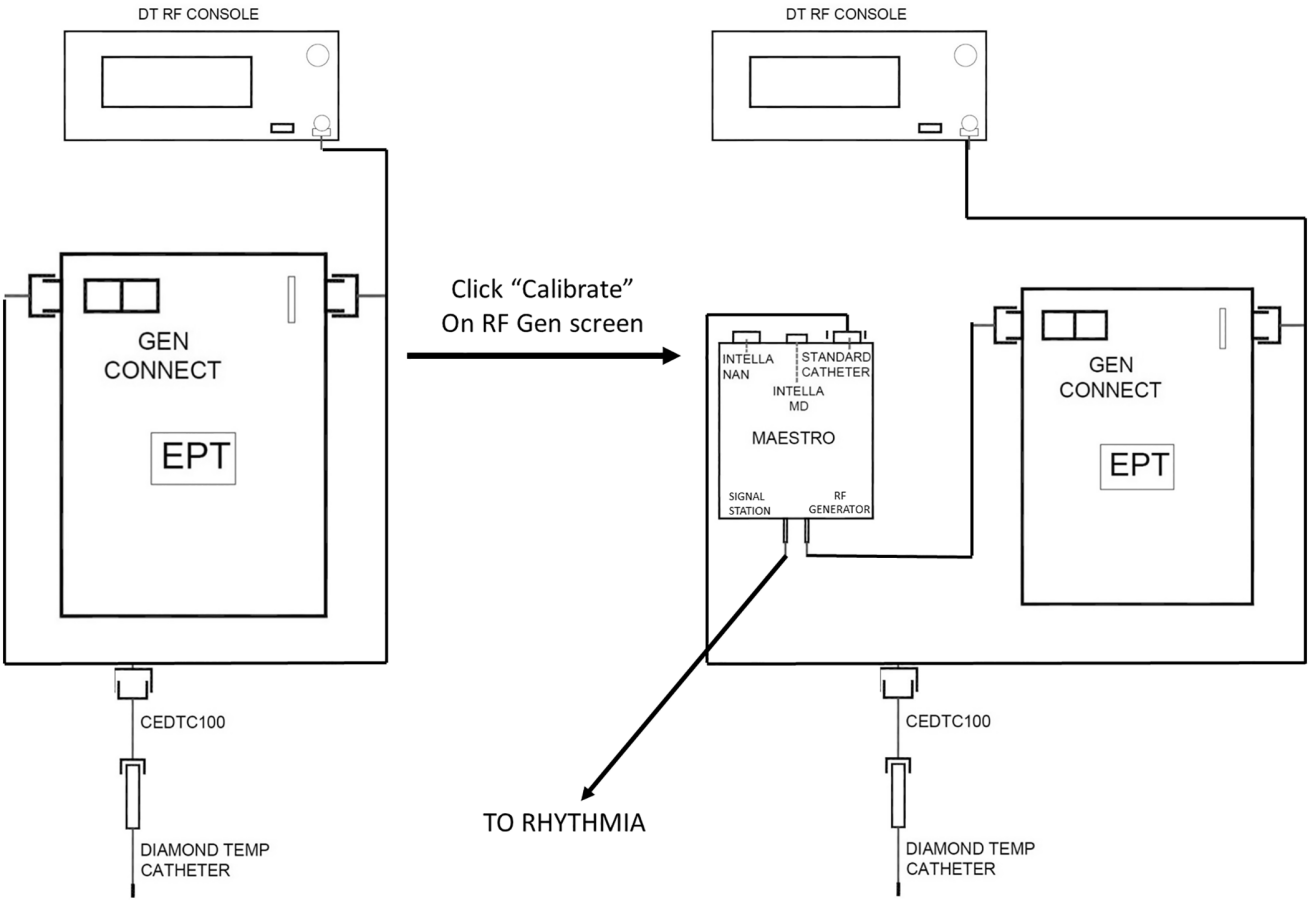
Schematic representation of the connections between DiamondTemp catheter and Rhythmia mapping system following calibration and prepared for use.

To allow visualization of the DT catheter while connected to the RF generator, calibration and connection were performed as follows: first, the DT catheter was connected to the GenConnect connection box (St Jude Medical) and, from it, to a dedicated RF generator (Epix Therapeutics, Inc.); second, calibration was performed; third, the Maestro ablation box (Boston Scientific) was connected between the DT catheter and the GenConnect box (Figure 1).

### 2.3 Ablation settings

Ablation parameters were set as per manufacturer recommendations: temperature‐controlled ablation limited to 60°C with a maximum power of 50 W. The time limit for RF application was left at nominal settings (20 s). RF applications were stopped after 10 s when the temperature reached >50°C and an impedance drop of at least 15% was observed after a minimum of 8 s, as indicative of a good RF delivery. RF was stopped prematurely if the temperature did not reach 50°C in the first 5 s. Lesion tags were manually placed upon adequate RF lesions with catheter stability.

### 2.4 Performance measuring

**FIGURE 2:**
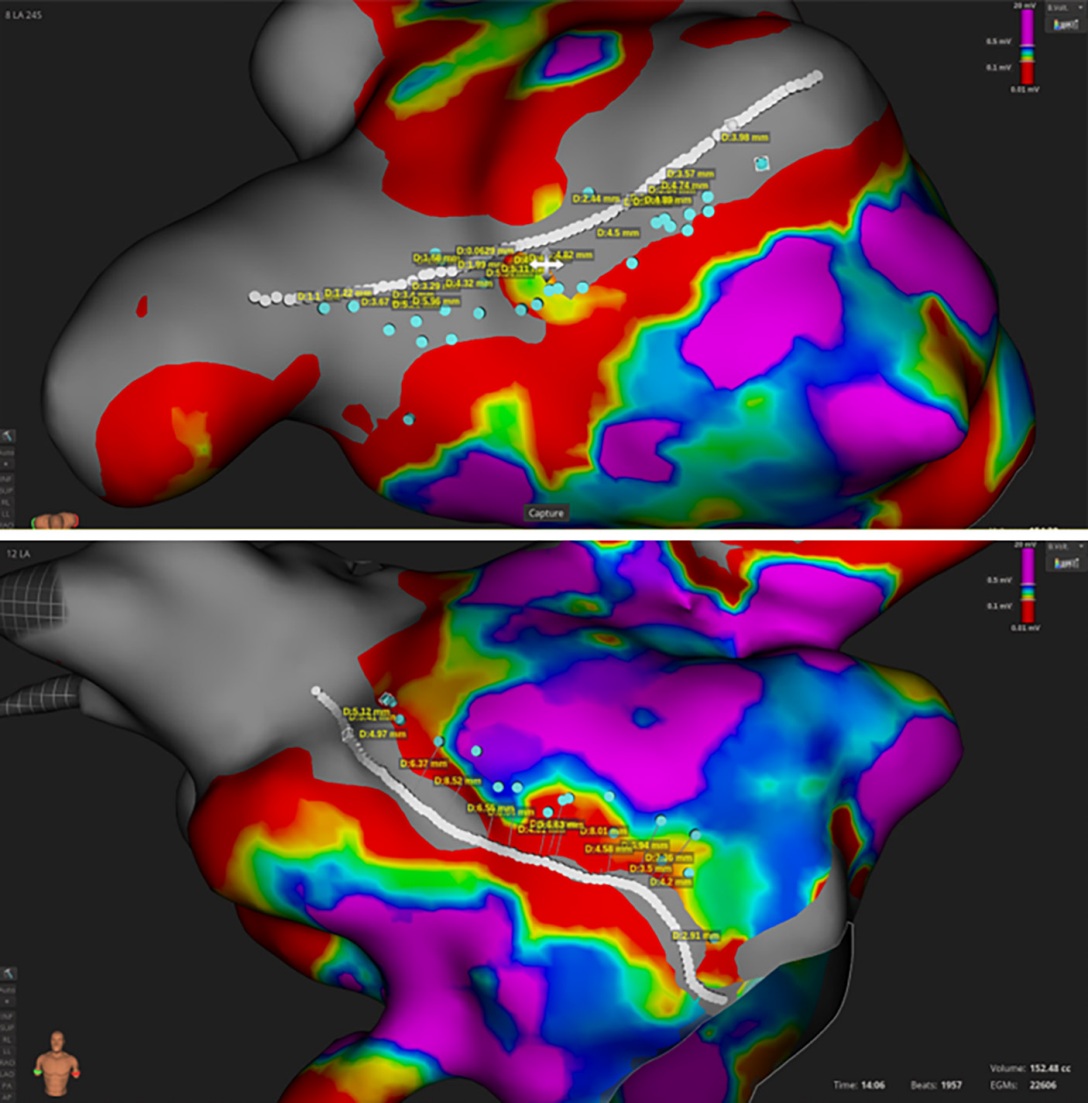
Performance measurements: ablation lesions (blue dots) and calculated distance to the line that represents the center of the scar (white). Upper panel: measurements performed on a roof line. Lower panel: measurements performed on an anteroseptal mitral line.

After the lesion set was completed, voltage remap was performed using the IntellaMap Orion catheter under stimulated atrial rhythm (scale adjusted to 0.05–0.1 mV). The center of the low‐voltage areas corresponding to previously performed ablation lines was taken as a reference point. Distance from this reference to the ablation tags—projected on the new map and reduced from 2.5 to a 0.5 mm radius to maximize the precision of each measurement—and interlesion distance was measured as a primary and secondary performance endpoints, respectively (tag‐to‐center scar distance) (Figure 2).

Procedures were performed by four experienced operators. After every procedure, they were asked to rate their agreement with three aspects regarding visualization performance of DT catheter in Rhythmia on a 0–5 Likert scale.

## 3 RESULTS

Fifty‐four left and right atrial maps were performed in 20 patients, and 1.778 ablation RF lesions were performed to complete 62 lines. Noninducibility was achieved in 85% of the patients, after treating 2.75 ± 1.16 MRAT per patient. The mean RF time per lesion was 13.3 ± 1.65 s, with an average of 19.7 ± 9.2 min of RF per complete lesion set.

Postablation remaps were performed in 17 patients, and 711 ablation tags from 40 lines were analyzed. Finally, tags related to four lines were excluded due to insufficient point density in the putative scar area during the remap. Overall, mean tag‐to‐center scar distance was 3.88 ± 2.95 mm. Data regarding the distribution of distances depending on the type of line is summarized in Table 1.

**TABLE 1:** Tag‐to‐center scar distance classified by type of ablation line.

	Tag‐to‐center scar distance		Interlesion distance	
	Mean	SD	Mean	SD
Average (all lesions)	3.88	2.95		
Right pulmonary veins (RPV) encircling	4.10	3.38	4.58	2.68
Left pulmonary veins (LPV) encircling	3.91	2.84	4.14	2.37
Anteroseptal left atrium (LA) line	3.46	2.62	3.61	1.94
Mitral isthmus line	3.78	2.36	2.88	1.74
Roofline	4.29	3.19	3.88	1.92
Lateral right atrium (RA) line	3.89	2.76	3.72	2.40
Cavotricuspid isthmus			2.95	1.46

Operator satisfaction with the use of DT in the Rhythmia HDx environment is displayed in Table 2. The reported user experience was overall satisfactory, reporting similar behavior as with the standard settings.

**TABLE 2:** Likert scale results show the operators' impression of catheter visualization performance.

Statement	Strongly disagree	Disagree	Neutral	Agree	Strongly agree
The relationship between catheter motion and visualization response in the mapping system is poorer in comparison with your prior experience with the Boston Scientific IntellaNav MiFi ablation catheter	18	2	0	0	0
Catheter visualization showed irregularities during navigation or ablation	19	1	0	0	0
The relationship between catheter projection on the map and the quality of the RF lesion (temperature rise, impedance drop) was strong	0	0	0	6	14

Abbreviation: RF, radiofrequency.

## 4 CONCLUSION

Visualization of the DT catheter on Rhythmia HDx mapping system is feasible and accurate, with no impact on workflow or acute procedural endpoints. Deviations between lesion tags and effective ablation lesions are within the range of lesion size and therefore should not affect the contiguity of ablation lines.

## ACKNOWLEDGEMENTS

There was no funding source for this particular study.

## CONFLICT OF INTEREST STATEMENT

Daniel Rodriguez Muñoz and Fernando Arribas Ynsaurriaga have received speaker honoraria and research funding from Medtronic, Inc. The rest of the authors have no conflict of interest to declare.

## DECLARATIONS

*Approval of the research protocol*: The protocol was approved by the ethics committee at University Hospital 12 de Octubre in December 2019, protocol number 20/274. *Informed consent*: All patients signed informed consent. *Registry and registration no*: University Hospital 12 de Octubre, protocol number 20/274. *Animal studies*: N/A.
